# Standardized uptake values in FDG PET/CT for prosthetic heart valve endocarditis: a call for standardization

**DOI:** 10.1007/s12350-017-0932-x

**Published:** 2017-06-05

**Authors:** Asbjørn M. Scholtens, Laurens E. Swart, Henryk J. te Kolste, Ricardo P. J. Budde, Marnix G. E. H. Lam, Hein J. Verberne

**Affiliations:** 1 Department of Nuclear Medicine, Meander Medical Center, Maatweg 3, 3813TZ Amersfoort, The Netherlands; 2 Department of Radiology and Nuclear Medicine, Erasmus Medical Center, Rotterdam, The Netherlands; 3 Department of Cardiology, VU Medical Center, Amsterdam, The Netherlands; 4 Department of Radiology and Nuclear Medicine, University Medical Center, Utrecht, The Netherlands; 5 Department of Radiology and Nuclear Medicine, Academic Medical Center, Amsterdam, The Netherlands

**Keywords:** ^18^F-fluorodeoxyglucose, positron emission tomography, computed tomography, prosthetic heart valve, endocarditis, EARL

## Abstract

**Background:**

The significance of and threshold values for the standardized uptake value (SUV) in FDG PET/CT to diagnose prosthetic heart valve (PHV) endocarditis (PVE) are unclear at present.

**Methods:**

A literature search was performed in the PubMed and EMBASE medical databases, comprising the following terms: (FDG OR *fluorode* OR *fluoro-de*) AND (endocarditis OR prosthetic heart valve OR valve replacement). Studies reporting SUVs correlated to the diagnosis of PVE were selected for analysis.

**Results:**

8 studies were included, with a total of 330 PHVs assessed. SUVs for PVE varied substantially across studies due to differences in acquisition, reconstruction, and measurement protocols, with median SUVmax values for rejected PVE ranging from 0.5 to 4.9 and for definite PVE ranging from 4.2 to 7.4.

**Conclusion:**

Reported SUV values for PVE are not interchangeable between sites, and further standardization of quantification is desirable. To this end, optimal protocols for patient preparation, image acquisition, and reconstruction and measurement methods need to be standardized across centers.

**Electronic supplementary material:**

The online version of this article (doi:10.1007/s12350-017-0932-x) contains supplementary material, which is available to authorized users.

## Introduction

In recent years, ^18^F-fluorodeoxyglucose positron emission tomography with computed tomography-based attenuation correction (FDG PET/CT) has been used increasingly in the setting of infection detection in general and prosthetic heart valve (PHV) endocarditis (PVE) in particular. FDG PET/CT has been proposed as a new criterion for the modified Duke classification^1^ and has been added to the European Society of Cardiology guidelines for the diagnosis and management of infective endocarditis.^2^

Most of the available studies on FDG PET/CT for PVE focus on the visual interpretation of images to differentiate between normal and pathological findings. FDG PET/CT is also able to semi-quantitatively measure the amount of metabolic activity of a lesion in the form of the standardized uptake value (SUV). This concept is appealing since it might offer objective cut-off values to discriminate normal from pathological uptake levels, relying less on subjective interpretation. However, SUV is dependent on a large number of variables regarding acquisition and reconstruction parameters, rendering the true applicability of the term “standardized” somewhat questionable.

Further standardization of the SUV has been proposed in a number of ways,^3^,^4^ including the European Association of Nuclear Medicine Research Ltd. (EARL) accreditation.

We performed a review of the available literature to ascertain whether a range of normal values for FDG PET/CT for PVE could be established.

## Methods

A literature search was performed in the PubMed and EMBASE medical databases, comprising the following terms: (FDG OR *fluorode* OR *fluoro-de*) AND (endocarditis OR prosthetic heart valve OR valve replacement). Search results were screened to comply with the following predetermined criteria:

- English language only
- No single case reports, case series acceptable
- Patient group with cardiac valve replacement
- SUV values reported as median and ranges or individual values and compared to diagnosis.

Eligible articles were read in full by one researcher (AMS) and their references screened for possible additional studies which fit the criteria, but none were found.

## Results

**Figure 1:**
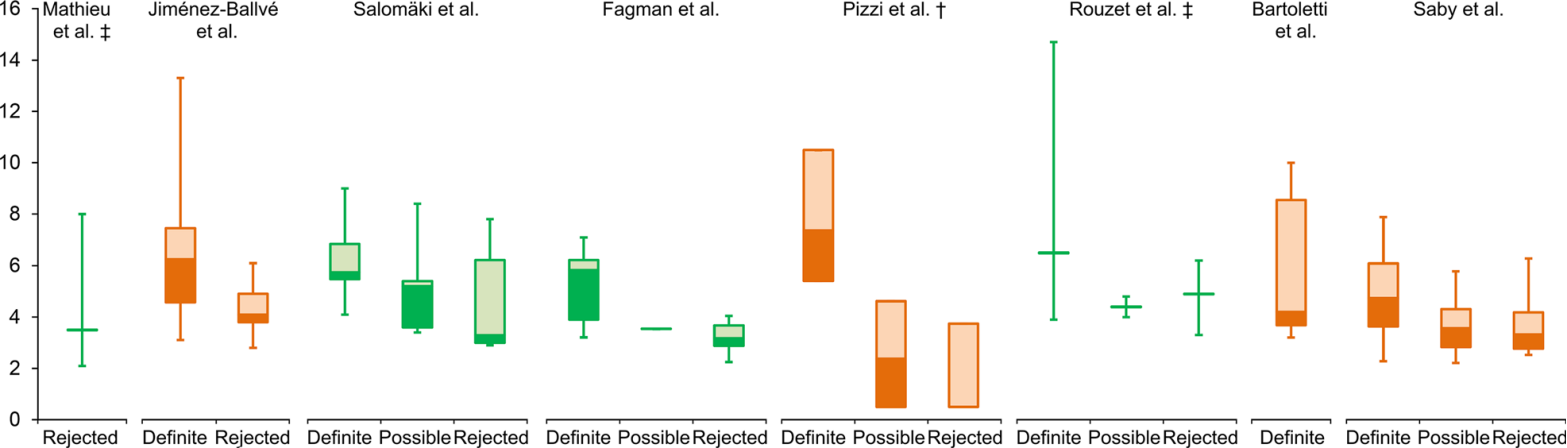
Reported SUVs in the eligible studies as median, interquartile ranges and total ranges unless noted otherwise. *Green values* were reported by EARL-accredited centers. ^‡^Values reported as median with total ranges. ^†^Values reported as median with quartile ranges

Out of 154 results of our initial literature search, 8 studies were found to be eligible under the predetermined criteria.^1^,^5^-^11^ Four studies were performed on EARL-accredited systems.^6^,^8^,^9^,^11^ In total, 330 PHVs were assessed. Study characteristics are described in Table 1 and boxplot representations of the SUVmax findings for each study are shown in Figure 1.

**Table 1:** **Study characteristics**

Study	Year	Patients	Valves	Patient preparation	FDG dose	Time to acquisition	Reconstruction method reported	EARL-accredited site	Camera type
Mathieu et al	2017	51	54	6 hour fast*	4 MBq/kg	60 minute	3-dimensional time-of-flight ordered-subsets expectation maximization	Yes	GE discovery 690
Jiménez-Ballvé et al	2016	41	42	48 hour HFLC diet, 12 hour fast, 50 IU/kg heparin pre-administration	5 MBq/kg	45–60 minute	Iterative reconstruction	No	Siemens biograph 6 TruePoint
Salomäki et al	2015	23	16	24 hour LC diet, 10 hour fast	mean 280 MBq	mean 72 minute	Ordered subset expectation maximization	Yes	GE discovery VCT
Fagman et al	2015	30	30	HFLC meal, 18 hour fast (N = 11)	4 MBq/kg	60 minute	Default iterative	Yes	Siemens biograph TruePoint 64
				6 hour fast (N = 19)^†^					
Pizzi et al	2015	92	65	12 hour fast, 50 IU/kg heparin pre-administration	3.7 MBq/kg	60 minute	Iterative TrueX + Time-of-Flight	No	Siemens biograph mCT 64S
Rouzet et al	2014	39	45	HFLC meal, 12 hour fast	4 MBq/kg	60 minute	Iterative 3-dimensional reconstruction algorithm of the system software	Yes	GE discovery 690
Bartoletti et al	2014	6	6	*Not reported*	*Not reported*	*Not reported*	*Not reported*	No	*Not reported*
Saby et al	2013	72	72	HFLC meal, 12 hour fast	5 MBq/kg	60 minute	Ordered subset expectation maximization	No	GE discovery ST

* Scans with intense, homogenous uptake in myocardium excluded from analysis

^†^ Controls based on oncologic scan protocols

*HFLC*, high fat, low carbohydrate; *LCD*, low carbohydrate diet; *IU*, International Units; *MBq*, Megabecquerel

In the seminal publication by Saby et al,^1^ 72 patients suspected of PVE were prospectively included. Findings on FDG PET/CT were compared to the final diagnosis, defined according to the modified Duke criteria after a follow-up period of 3 months. Visual analysis was based on hypermetabolism in prosthetic and periprosthetic areas on both attenuation-corrected (AC) and non-AC (NAC) images. SUVmax was measured as the average of 3 measurements from 3 volumes of interest (VOI) of 5 mm^3^ at equal distances from each other. Additionally, a 35 mm^3^ VOI was placed in the right atrium blood pool in a location without significant metabolic activity from myocardium, and target-to-background ratio (TBR) was calculated as (SUVmax valve/SUVmax atrial blood pool). The reported sensitivity, specificity, positive predictive value (PPV), negative predictive value (NPV), and global accuracy for visual assessment were 73%, 80%, 85%, 67%, and 76%, respectively. By adding FDG PET/CT to the modified Duke criteria, sensitivity increased to 97%.

Bartoletti et al^5^ only included patients with proven PVE in their case series of 6 patients. They did not describe the measurement method for the reported values. They found a large variation in SUVmax, with several relatively low values (median 4.2, range 3.2-10.0). In patients with the lowest values, antibiotic therapy had been started before FDG PET/CT with resolution of fever and other symptoms of infection.

Rouzet et al^6^ included 39 patients with a total of 45 PHVs in their study on FDG PET/CT and radiolabelled leukocyte scintigraphy in PVE. Visual analysis and SUV measurement were performed based on the same methods as reported by Saby et al and final diagnosis was also based on modified Duke criteria after 3 months follow-up. The reported sensitivity, specificity, PPV, NPV, and global accuracy for visual assessment were 93%, 71%, 68%, 94%, and 80% respectively. SUVs for patients classified as ‘rejected’ by the modified Duke criteria were relatively high (median 4.9, range 3.3-6.2), but patients with no visually discernible uptake of FDG in the region of the prosthetic valve were excluded from the semi-quantitative analysis. Since it is reasonable to assume that these would have represented lower SUVmax values, the reported SUVmax values will therefore most likely be skewed to the higher end of the spectrum.

The study by Pizzi et al^7^ included both patients with PHVs and cardiac implantable electronic devices (CIEDs). The reported sensitivity, specificity, PPV, and NPV for the total group were 84%, 75%, 81%, and 78%, respectively when compared to the modified Duke criteria at 3 months. For the purpose of this review only the values of the patients with PHVs were included. Visual analysis was comparable to the methods outlined by Saby et al, but SUV analysis differed in that SUVmax was measured at any abnormal area, and blood pool measurement was based on the mean standardized uptake value (SUVmean) as obtained with a 30 mm^3^ VOI at the thoracic descending aorta. TBR was calculated as (SUVmax prosthesis/SUVmean blood pool). The region of interest in scans without visually detectable uptake near the prosthetic valve was placed around the metallic components of the valve alone, without inclusion of the adjacent tissues and blood pool, which most likely resulted in a lower SUVmax and may explain the exceptionally low values found in the ‘rejected’ category (median 0.5).

Fagman et al^8^ included 11 patients scanned for suspected PVE. Additionally, 19 normal controls were added in the form of patients with prosthetic heart valves scanned for malignancy. Visual analysis, comparable to the methods described by Saby et al, resulted in a sensitivity of 75% and specificity of 84%, based on patients with definite (N = 8) or without (N = 19) PVE. Semi-quantitative analysis was performed measuring SUVmax in or directly adjacent to the prosthetic aortic valve. Blood pool values were determined by measuring SUVmax in five circular ROIs on consecutive slices in the lumen of the descending aorta at the level of carina, avoiding inclusion of potential uptake in the wall of the aorta. TBR was calculated as (prosthetic valve SUVmax/SUVmax descending aorta).

In their study, Salomäki et al^9^ included both native valves and PHVs suspected of endocarditis, confirming that FDG PET/CT is less capable of diagnosing native valve endocarditis (only 1 out of 6 cases detected). For the purpose of this review only the data of the 16 patients with PHVs were considered. Visual analysis was performed as described by Saby et al, resulting in a reported sensitivity of 100% (6/6 cases) but a specificity of only 60%. SUVmax was measured in a VOI covering the valve or prosthesis area based on co-registered CT images. The mean blood pool values were measured in the ascending aorta excluding the vessel wall (mean radioactivity in a VOI of 6.8 cm^3^) to calculate TBR. Two noticeably high values (7.8 and 7.2) skewed the overall findings in the ’rejected’ category (N = 5) upwards (median 4.8, range 2.9-7.8), with one value reported as being this high due to a foreign body reaction and one due to imaging relatively early after implantation (6 weeks).

Jiménez-Ballvé et al^10^ compared different interpretation criteria in their cohort consisting of patients with PHVs and/or CIEDs compared to the Duke pathological criteria if tissue was available or the decision of an endocarditis expert team after a minimum of 4 months follow-up. Using criteria comparable to those used by Saby et al in the whole group, visual analysis resulted in reported sensitivity, specificity, PPV, NPV, and global accuracy of 100%, 73%, 80%, 100%, and 87%, respectively. Again, for this review only the data in PHV patients compared to the final diagnosis were included. SUVmax was measured in the area under suspicion and was compared to physiological uptake (also measured as SUVmax) in the mediastinal blood pool, calculated by measuring a VOI with 3 mm diameter in the ascending aorta, and in the liver, calculated by measuring a VOI with 3 cm diameter drawn in the right hepatic lobe excluding any areas of inhomogeneous or focally increased uptake. As in the study by Rouzet et al, patients with no visually discernible uptake of FDG in the region of the prosthetic valve were excluded from the semi-quantitative analysis, probably skewing reported SUVmax values to the higher end of the spectrum.

Mathieu et al^11^ recently studied 51 patients with 54 PHVs with no suspicion of PVE to define normal variants and values of FDG uptake. Indications for PET were oncology (N = 26), suspicion of prosthetic valve endocarditis subsequently excluded (N = 17), and history of vasculitis (N = 11). Visual analysis was descriptive, with FDG uptake described as absent, homogeneous, or focal in 13%, 80%, and 7% of AC images and 44%, 50%, and 6% of NAC images, respectively. SUV measurements were performed according to the protocol as described by Saby et al and Rouzet et al, resulting in a median SUVmax of 3.5 (range 2.1-8.0). In subgroup analysis, values were higher in patients referred with a history of vasculitis (median 4.7, range 3.0-8.0) than in the other patients referred for oncologic indications (median 3.3, range 2.1-5.7) or rejected PVE (median 3.5, range 2.1-4.7), even though metabolic activity in the wall of the ascending aorta did not differ significantly between groups.

## Discussion

In general, the included studies show that higher SUVs are reported for patients with PVE than those without. However, the great variations in median values and their ranges are a concern, and proof that reported values cannot heedlessly be extrapolated into clinical practice.

The numerous patient- and preparation-related variables that influence the uptake of FDG in the region of the heart make interpreting FDG PET/CT images in the setting of PVE a challenging task. The use of antibiotics or corticosteroids can lead to false-negative results, foreign body reactions may be falsely interpreted as infection, and other confounders may influence the interpretation as well.^12^ Knowing this, we cannot expect the SUV to be the only distinguishing variable. Nevertheless, there is much to be gained by performing FDG PET/CT in a uniform way with truly standardized SUV measurements. As our review of the literature shows, there is currently very little standardization of how FDG PET/CT is performed in PVE, both regarding acquisition and reconstruction protocols as well as the definition of the region in which the uptake is to be quantified.

Although the different studies may have used different criteria to define the diagnosis of PVE, this can be argued to be a lesser concern in the context of this article. Even if the diagnostic criteria had been perfectly equal across the studies, the reported values would still be incomparable due to the differences in methodology of measurement and parameters of acquisition.

EARL-accredited reconstruction is a logical step towards better reproducibility of reconstruction parameters. The SUVref method, in which camera- and reconstruction-specific filter parameters are applied to produce images with standardized properties for SUV measurements^4^ is another option, which may have the added benefit of being applicable to non-EARL-accredited sites in retrospect.

The potential negative effect of EARL-accredited reconstructions is its relatively high level of smoothing of images, resulting in lower reported SUVmax measurements than on images based on contemporary reconstruction methods incorporating Time-of-Flight and other parameters, especially in the higher ranges. For this reason it may be desirable to add EARL-reconstructions only for the purpose of standardized measurements, using vendor- and camera-optimized reconstructions for visual analysis and clinical implementation.

To be able to reliably measure values in the region of PHVs, it is important that the physiological glucose metabolism (and by proxy FDG uptake) of the myocardium be suppressed. Many patient preparation protocols exist, with the optimal solution still up for debate.^13^ Based on our own experiences, a low carbohydrate fat-allowed diet for 24 hours, 12-hour fast and unfractionated heparin bolus pre-administration results in adequate suppression^14^ and shorter fasting periods should be avoided. Centers should strive for a protocol that reliably yields suppression in >80% of patients.^13^ Recently, Giorgetti et al published their findings on increased myocardial suppression in patients receiving low molecular weight heparin or warfarin therapy,^15^ and patients receiving such therapy likely do not need additional unfractionated heparin bolus administration.

Regarding measurement methods, a number of studies excluded valves with no visual abnormalities from semi-quantitative measurement, thus introducing a potential reporting bias in the values for normal valves and rejected PVE. As these excluded measurements likely had values close to blood pool values, the true median may be expected to be lower than reported. To reduce potential reporting bias we would recommend that SUV be measured and reported in all PHVs, not just in visually abnormal ones, with the same methodology used in all measurements. To improve ease of measurement (and hence reproducibility) we suggest reporting the SUVmax obtained from a single VOI encompassing the entire prosthesis where possible, rather than creating mean values from multiple SUVmax measurements in multiple VOIs.

By creating reproducible results, recommendations based on SUV measurements could become interchangeable between sites, and research data from various hospitals could safely be pooled into a larger dataset. The latter should be of particular interest, since the incidence of PVE is relatively low even in specialized centers. To allow for truly large studies with significant statistical power, it is inevitable to include multiple centers, which is inappropriate without better standardization of FDG PET/CT.

Once the process of FDG PET/CT imaging is comparable between sites, including every step from patient preparation to image interpretation and measurement, we can begin to understand the effects of the other variables based on reliable interpretation and measurement standards.

## New Knowledge Gained

SUVs for PVE reported in the literature are highly dependent on acquisition, reconstruction, and measurement protocols, and further standardization is needed before values are interchangeable between centers.

### Recommendations

**Figure 2:**
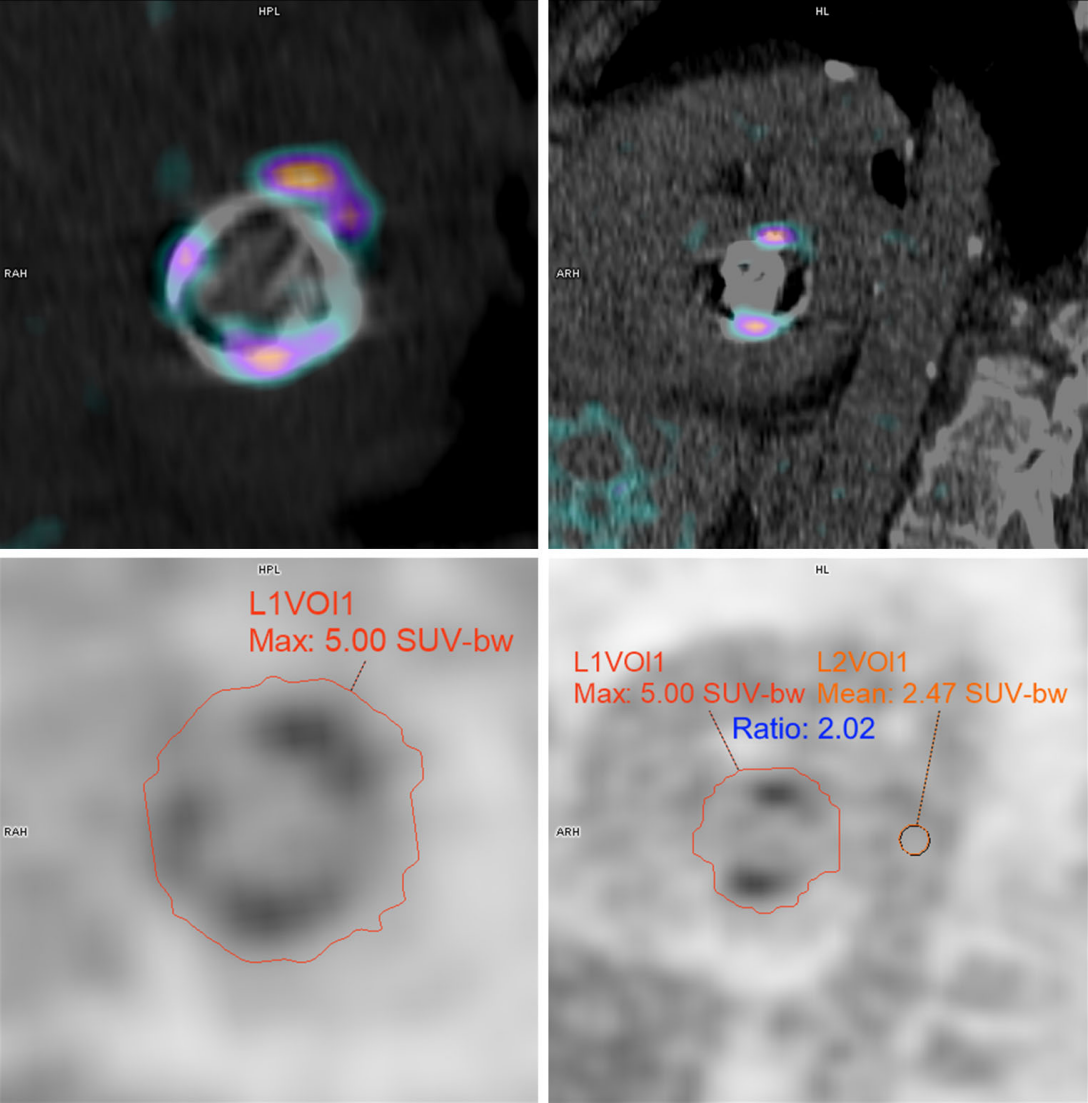
Example of proposed measurement standardization in a mitral valve prosthesis. Fused PET/CT images (*upper row*) and corresponding attenuation-corrected PET images (*below*) showing whole-valve measurement VOI (in this case a self-expanding VOI set to include voxels ≥40% of maximum) and VOI sphere in the descending aorta (*lower right*). Uptake in myocardium (hardly present here) and the aortic wall are excluded

To improve uniformity in measurements we recommend patient preparation with a carbohydrate-restricted diet and a prolonged fast coupled with heparin bolus administration preceding the administration of FDG.^14^ Reported measurements should be performed on reconstructions according to the EARL accreditation, and should include whole-valve or whole-prosthesis measurements of the SUVmax (with VOI excluding physiological myocardial uptake) as well as a measurement of the SUVmean of the blood pool in the descending aorta (VOI excluding vessel wall) to calculate a target-to-background ratio (example in Figure 2). VOI measurements of SUVmean in the right atrium and the liver may be of additional value to ascertain the optimal region for TBR calculation.

We believe the above recommendations will result in robust and reproducible measurements which will allow for cross-center comparability and pooling of results.

## Conclusion

SUVs reported in the current literature on FDG PET/CT in PVE vary according to acquisition, reconstruction, and measurement methods, emphasizing the need for a uniform protocol to allow for better comparison of results between different centers. Although not without drawbacks, standardized measurements on EARL-accredited reconstructions seem a sensible and feasible starting point.

## Electronic supplementary material

Below is the link to the electronic supplementary material.

Supplementary material 3 (PPTX 1306 kb)

## Abbreviations

FDG: ^18^F-fluorodeoxyglucose
PET: Positron emission tomography
CT: Computed tomography
PHV: Prosthetic heart valve
PVE: Prosthetic heart valve endocarditis
CIED: Cardiac implantable electronic device
SUV: Standardized uptake value
EARL: European Association of Nuclear Medicine Research Ltd
AC: Attenuation corrected/correction
NAC: Non-attenuation corrected
