# Supplementary material for: Standardized uptake values in FDG PET/CT for prosthetic heart valve endocarditis: a call for standardization
Source: J Nucl Cardiol. 2017 Jun 5;25(6):2084–91. doi: 10.1007/s12350-017-0932-x (PMC6280770; doi:10.1007/s12350-017-0932-x)
Supplement: Supplementary file 1 — Supplementary material 3 (PPTX 1306 kb) [file 12350_2017_932_MOESM1_ESM.pptx]

## Slide 1
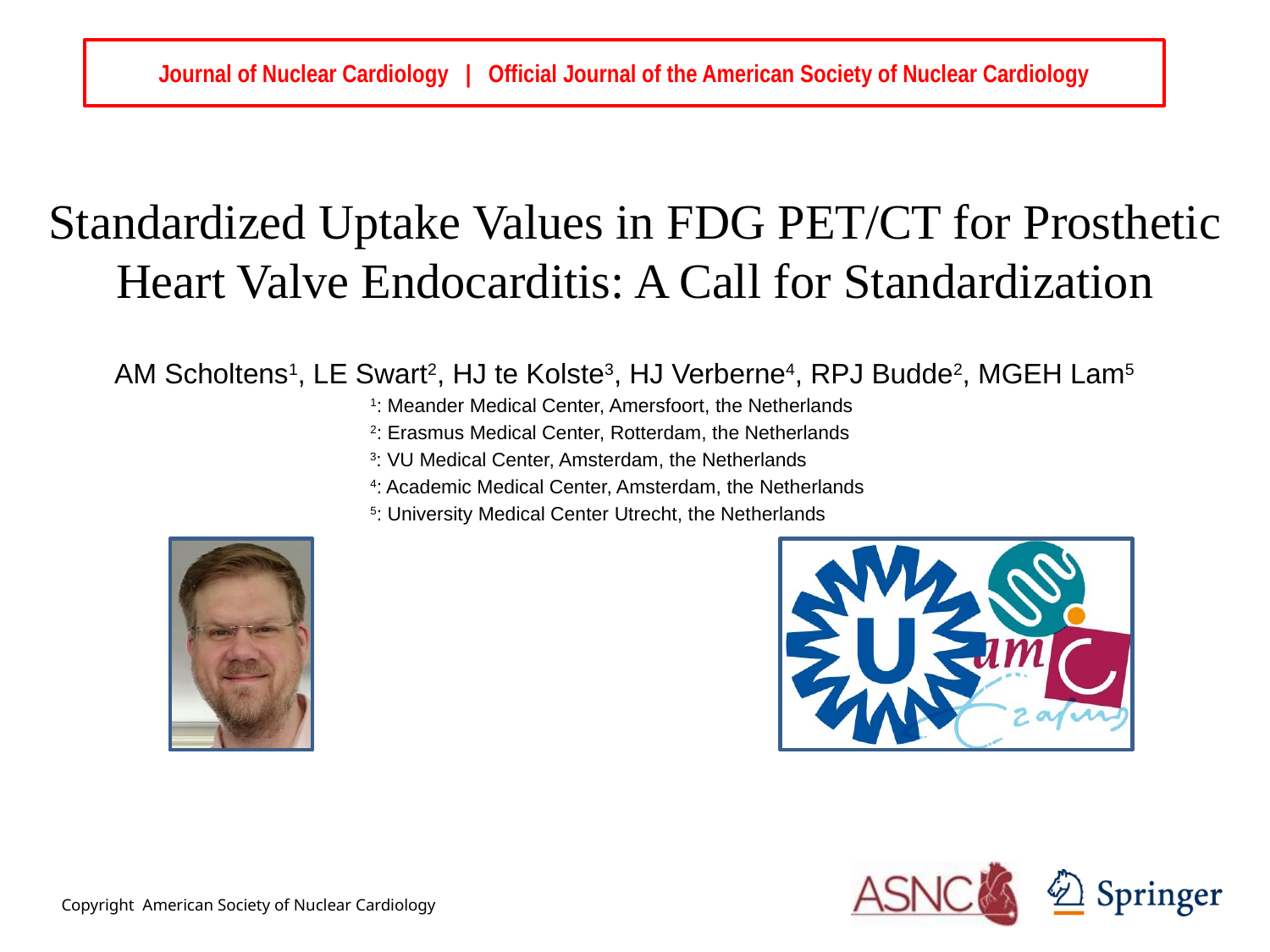

Journal of Nuclear Cardiology | Official Journal of the American Society of Nuclear Cardiology
# Standardized Uptake Values in FDG PET/CT for Prosthetic Heart Valve Endocarditis: A Call for Standardization
AM Scholtens1, LE Swart2, HJ te Kolste3, HJ Verberne4, RPJ Budde2, MGEH Lam5
1: Meander Medical Center, Amersfoort, the Netherlands
2: Erasmus Medical Center, Rotterdam, the Netherlands
3: VU Medical Center, Amsterdam, the Netherlands .
4: Academic Medical Center, Amsterdam, the Netherlands
5: University Medical Center Utrecht, the Netherlands
Copyright American Society of Nuclear Cardiology

## Slide 2
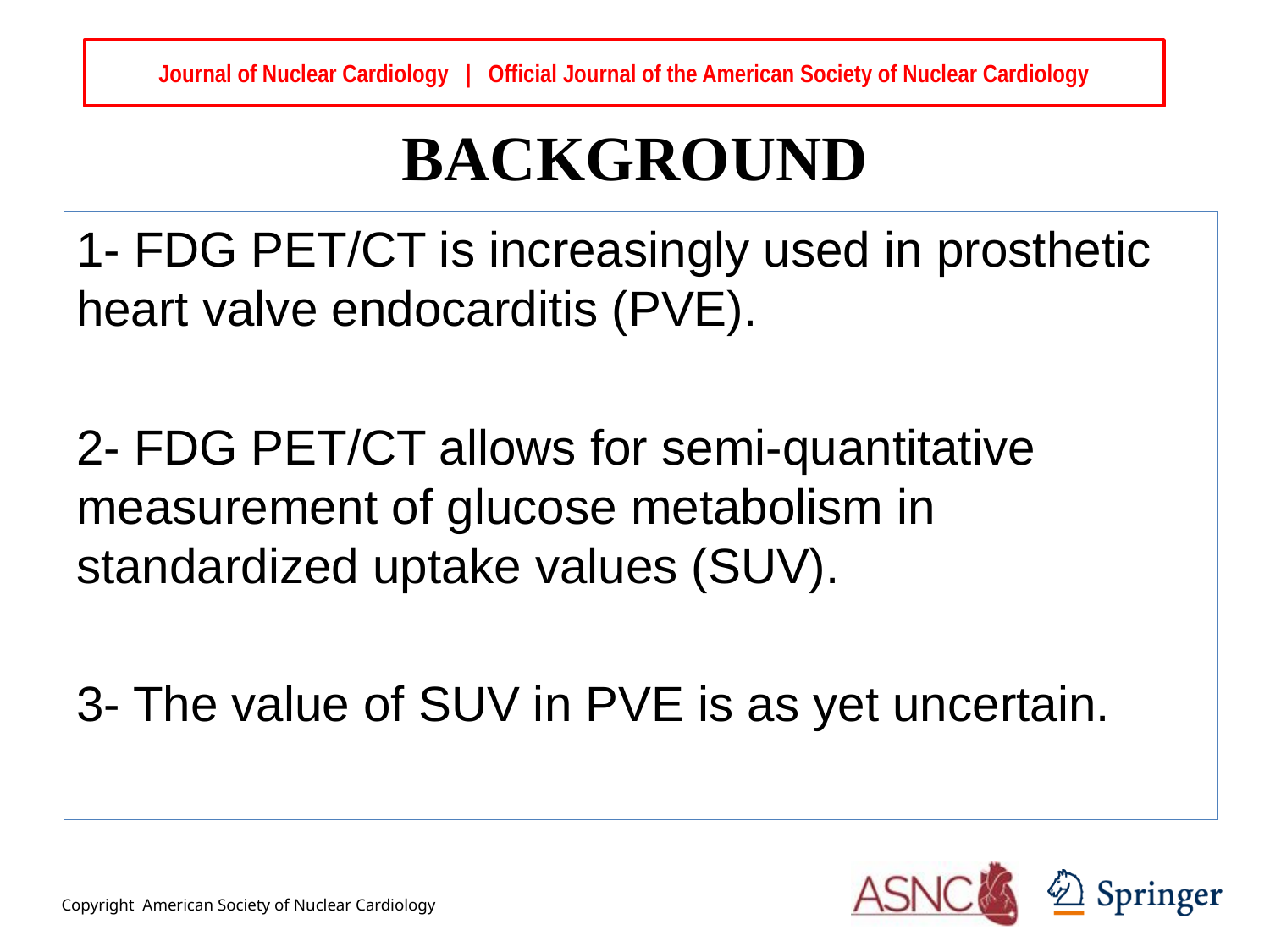

Journal of Nuclear Cardiology | Official Journal of the American Society of Nuclear Cardiology
# BACKGROUND
1- FDG PET/CT is increasingly used in prosthetic heart valve endocarditis (PVE).
2- FDG PET/CT allows for semi-quantitative measurement of glucose metabolism in standardized uptake values (SUV).
3- The value of SUV in PVE is as yet uncertain.
Copyright American Society of Nuclear Cardiology

## Slide 3
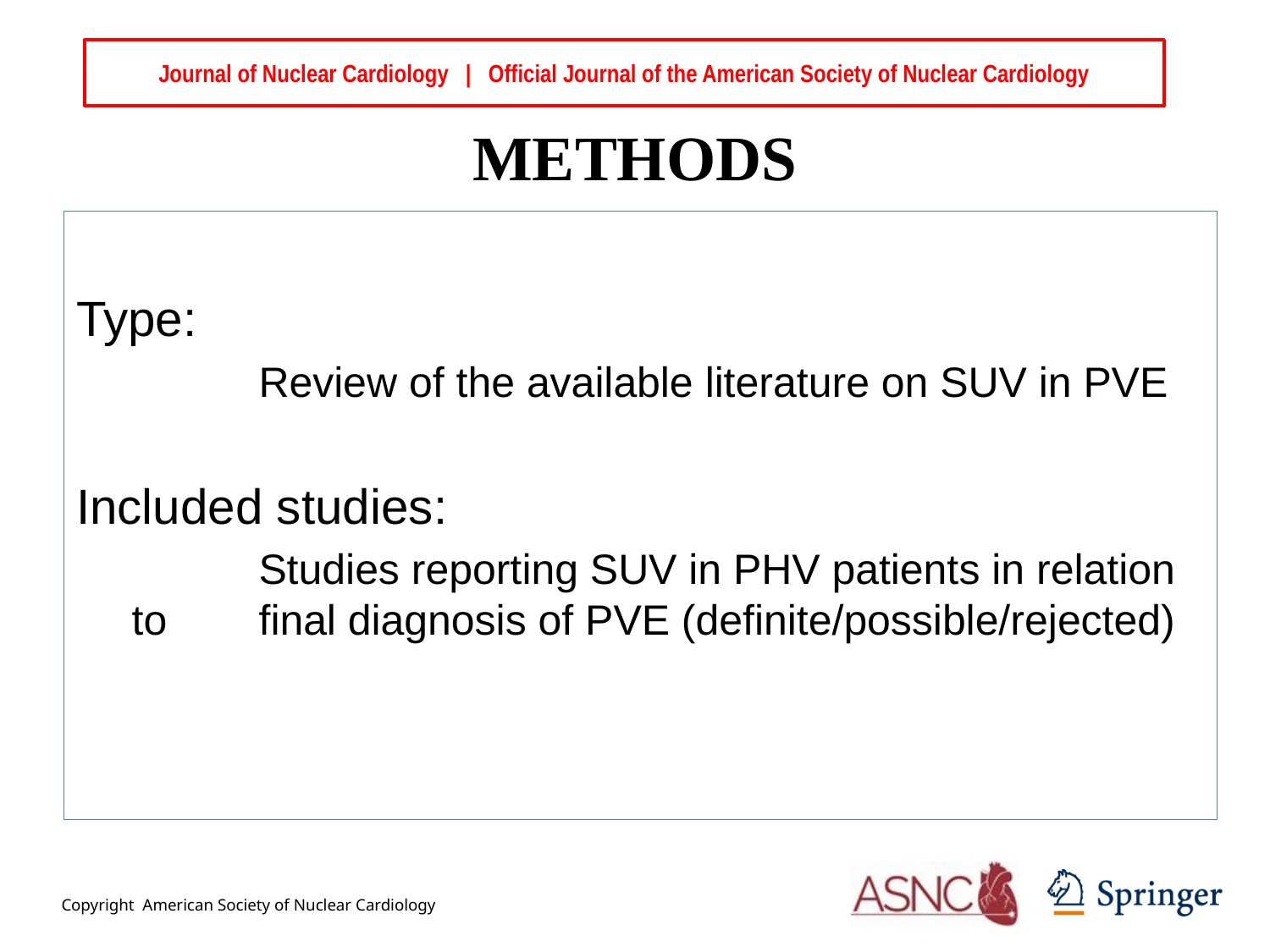

Journal of Nuclear Cardiology | Official Journal of the American Society of Nuclear Cardiology
# METHODS
Type:
	Review of the available literature on SUV in PVE
Included studies:
	Studies reporting SUV in PHV patients in relation to 	final diagnosis of PVE (definite/possible/rejected)
Copyright American Society of Nuclear Cardiology

## Slide 4
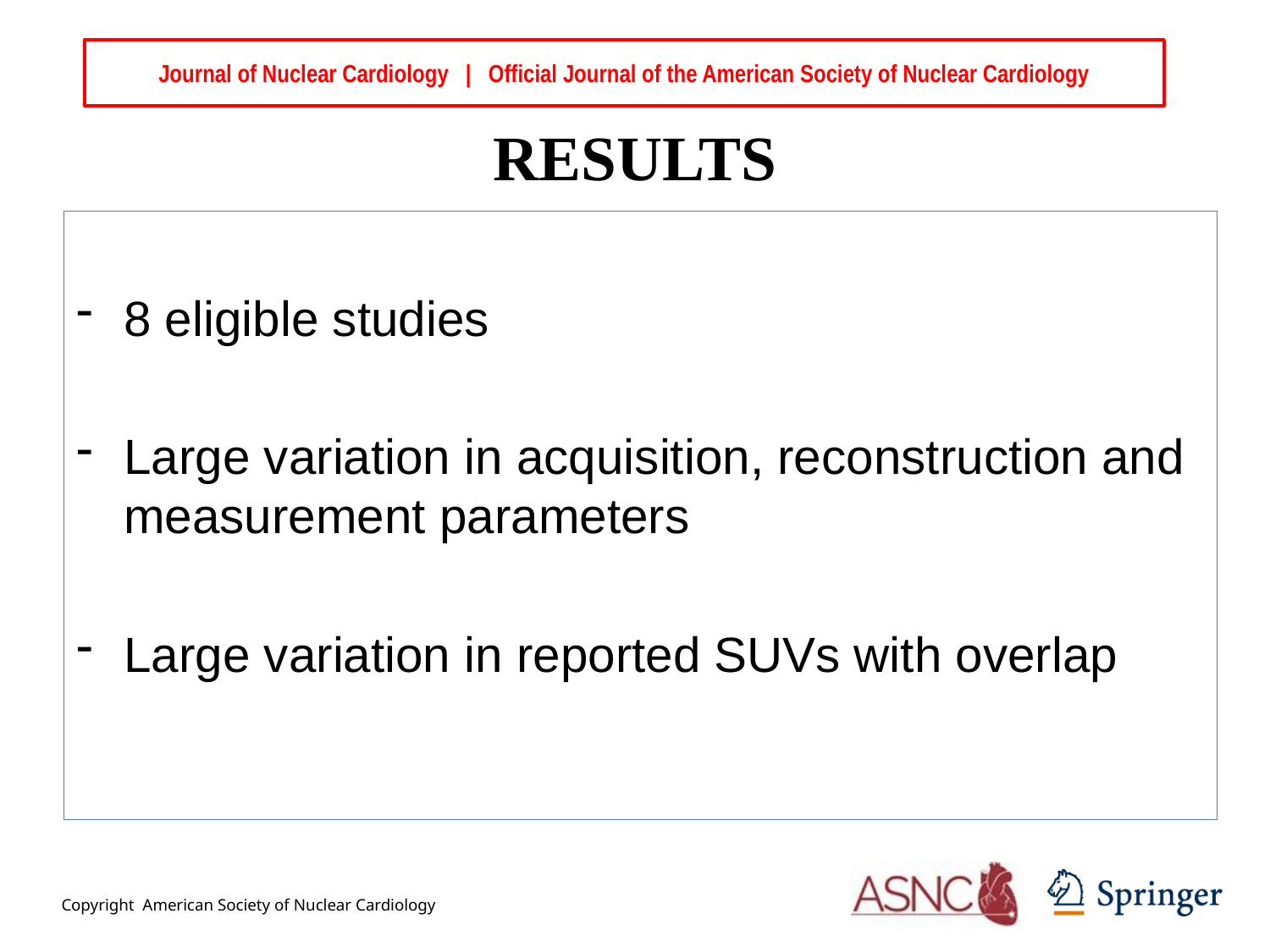

Journal of Nuclear Cardiology | Official Journal of the American Society of Nuclear Cardiology
# RESULTS
8 eligible studies
Large variation in acquisition, reconstruction and measurement parameters
Large variation in reported SUVs with overlap
Copyright American Society of Nuclear Cardiology

## Slide 5
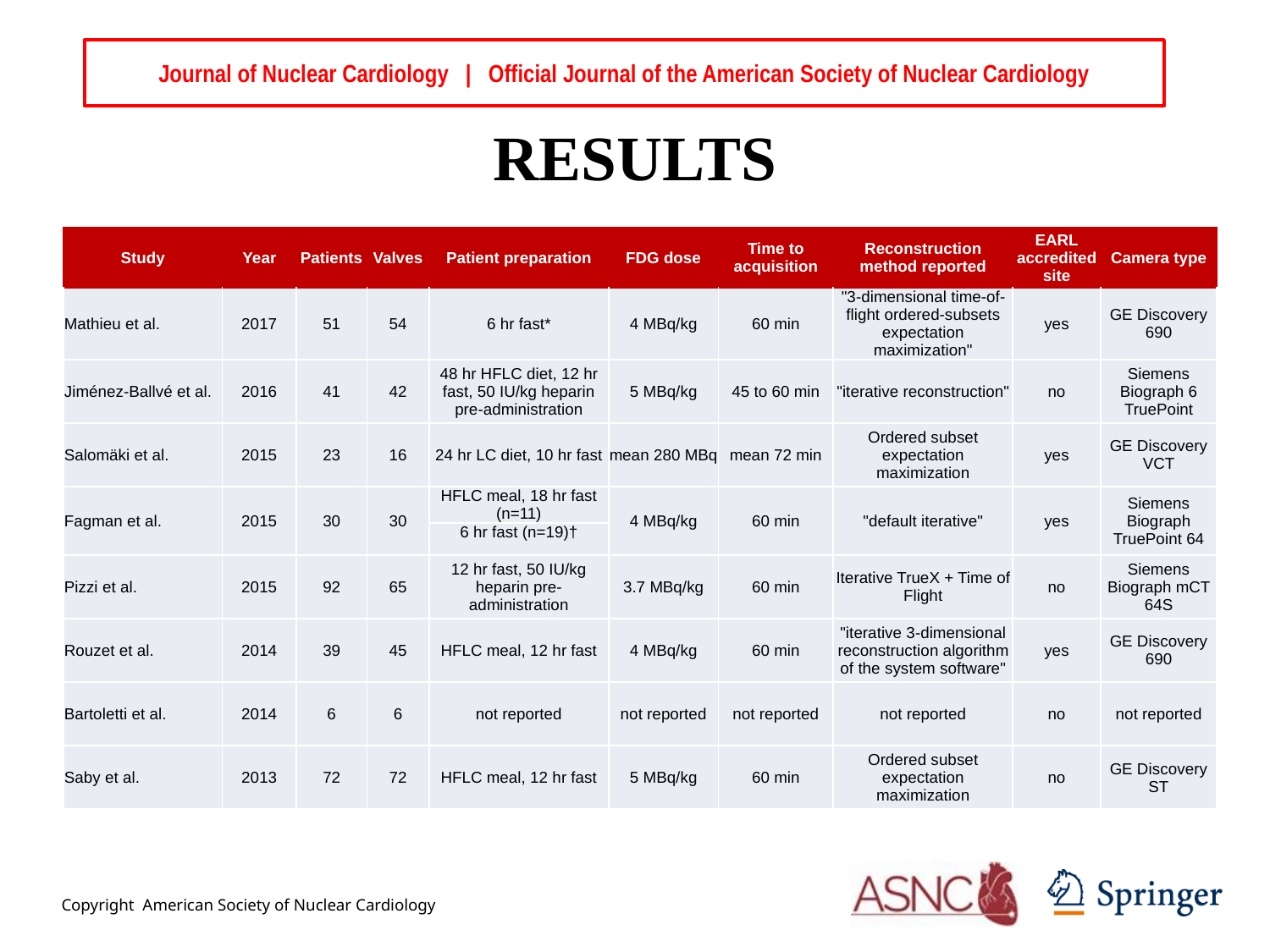

Journal of Nuclear Cardiology | Official Journal of the American Society of Nuclear Cardiology
# RESULTS
| Study | Year | Patients | Valves | Patient preparation | FDG dose | Time to acquisition | Reconstruction method reported | EARL accredited site | Camera type |
| --- | --- | --- | --- | --- | --- | --- | --- | --- | --- |
| Mathieu et al. | 2017 | 51 | 54 | 6 hr fast\* | 4 MBq/kg | 60 min | "3-dimensional time-of-flight ordered-subsets expectation maximization" | yes | GE Discovery 690 |
| Jiménez-Ballvé et al. | 2016 | 41 | 42 | 48 hr HFLC diet, 12 hr fast, 50 IU/kg heparin pre-administration | 5 MBq/kg | 45 to 60 min | "iterative reconstruction" | no | Siemens Biograph 6 TruePoint |
| Salomäki et al. | 2015 | 23 | 16 | 24 hr LC diet, 10 hr fast | mean 280 MBq | mean 72 min | Ordered subset expectation maximization | yes | GE Discovery VCT |
| Fagman et al. | 2015 | 30 | 30 | HFLC meal, 18 hr fast (n=11) | 4 MBq/kg | 60 min | "default iterative" | yes | Siemens Biograph TruePoint 64 |
| | | | | 6 hr fast (n=19)† | | | | | |
| Pizzi et al. | 2015 | 92 | 65 | 12 hr fast, 50 IU/kg heparin pre-administration | 3.7 MBq/kg | 60 min | Iterative TrueX + Time of Flight | no | Siemens Biograph mCT 64S |
| Rouzet et al. | 2014 | 39 | 45 | HFLC meal, 12 hr fast | 4 MBq/kg | 60 min | "iterative 3-dimensional reconstruction algorithm of the system software" | yes | GE Discovery 690 |
| Bartoletti et al. | 2014 | 6 | 6 | not reported | not reported | not reported | not reported | no | not reported |
| Saby et al. | 2013 | 72 | 72 | HFLC meal, 12 hr fast | 5 MBq/kg | 60 min | Ordered subset expectation maximization | no | GE Discovery ST |
Copyright American Society of Nuclear Cardiology

## Slide 6
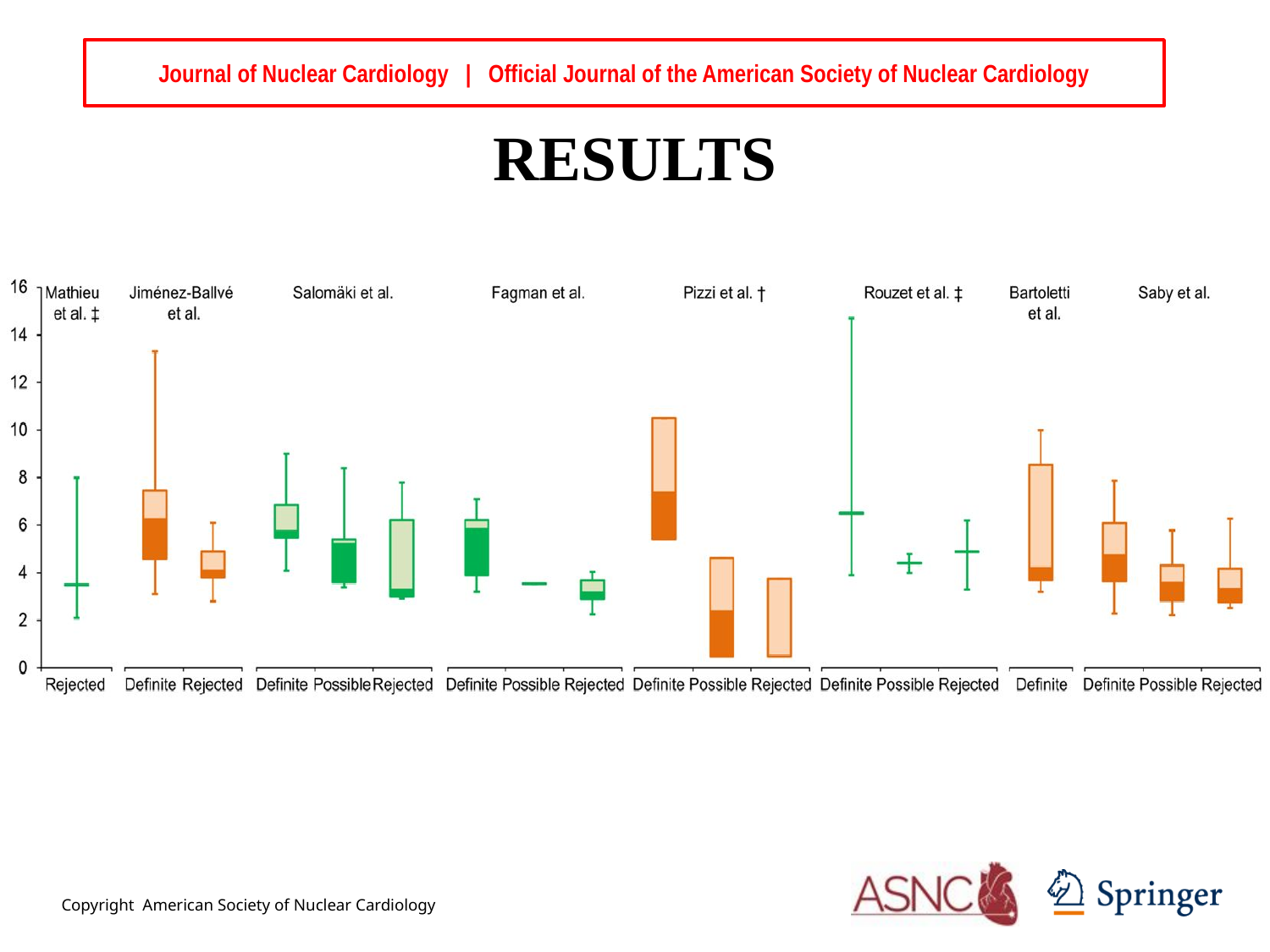

Journal of Nuclear Cardiology | Official Journal of the American Society of Nuclear Cardiology
# RESULTS
Copyright American Society of Nuclear Cardiology

## Slide 7
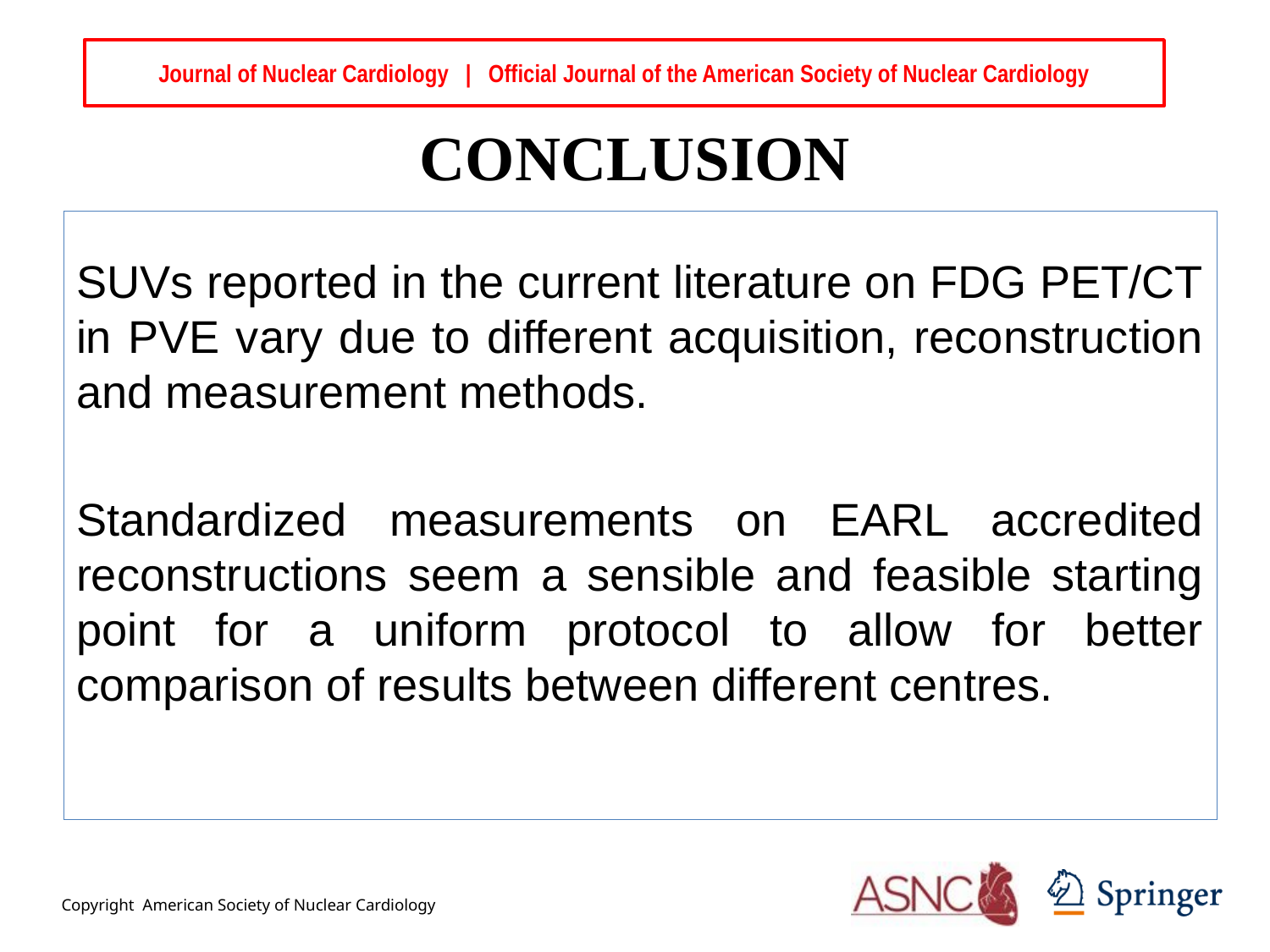

Journal of Nuclear Cardiology | Official Journal of the American Society of Nuclear Cardiology
# CONCLUSION
SUVs reported in the current literature on FDG PET/CT in PVE vary due to different acquisition, reconstruction and measurement methods.
Standardized measurements on EARL accredited reconstructions seem a sensible and feasible starting point for a uniform protocol to allow for better comparison of results between different centres.
Copyright American Society of Nuclear Cardiology

## Slide 8
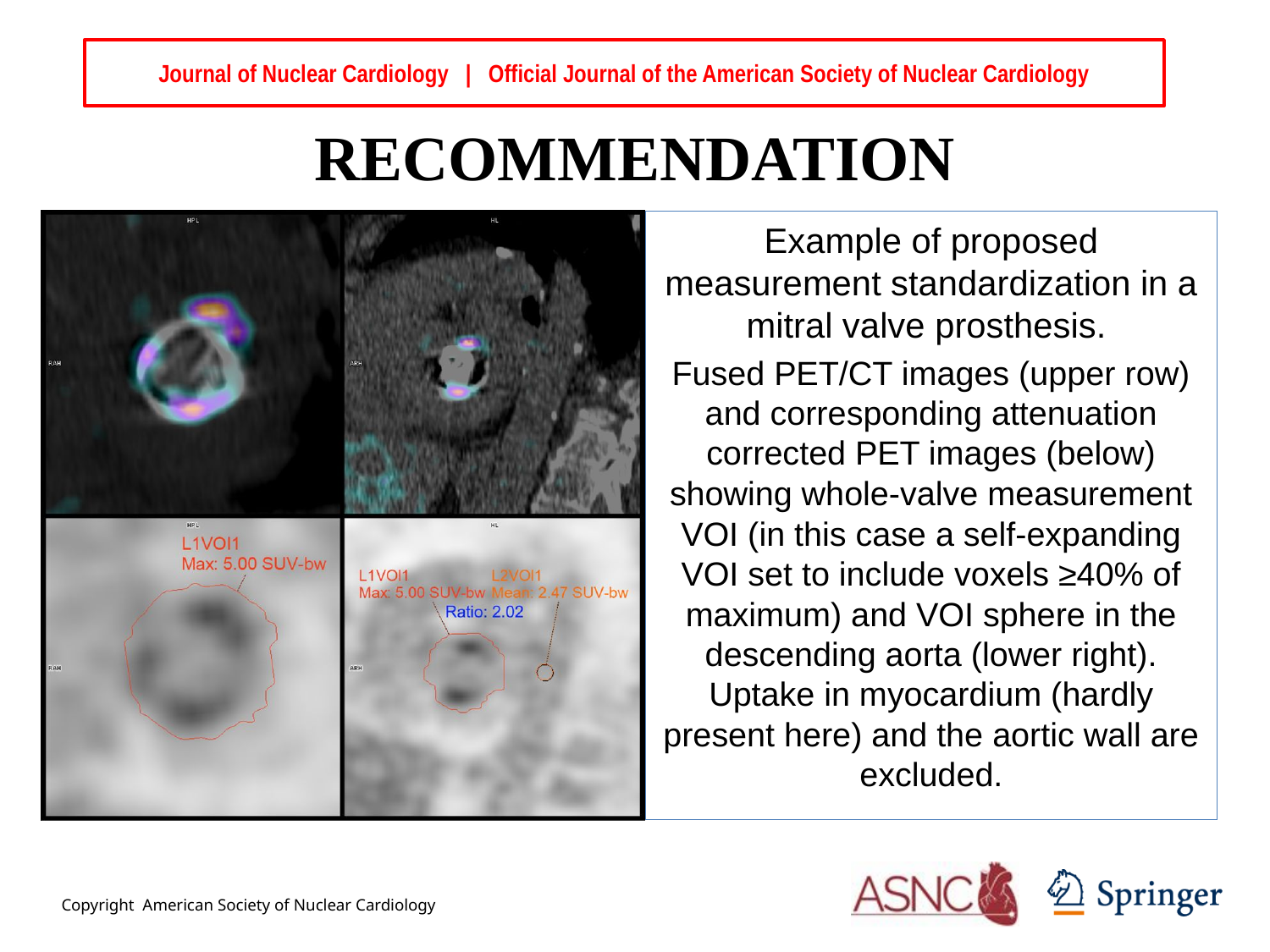

Journal of Nuclear Cardiology | Official Journal of the American Society of Nuclear Cardiology
# RECOMMENDATION
Example of proposed measurement standardization in a mitral valve prosthesis.
Fused PET/CT images (upper row) and corresponding attenuation corrected PET images (below) showing whole-valve measurement VOI (in this case a self-expanding VOI set to include voxels ≥40% of maximum) and VOI sphere in the descending aorta (lower right). Uptake in myocardium (hardly present here) and the aortic wall are excluded.
Copyright American Society of Nuclear Cardiology
